# Commentary: Mesenchymal Stem Cells: A New Piece in the Puzzle of COVID-19 Treatment

**DOI:** 10.3389/fimmu.2021.682195

**Published:** 2021-04-29

**Authors:** Juliana Lott Carvalho, Amandda Evelin Silva-Carvalho, Emãnuella Melgaço Garcez, Felipe Saldanha-Araujo

**Affiliations:** ^1^ Multidisciplinary Laboratory of Biosciences, Faculty of Medicine, University of Brasilia, Brasilia, Brazil; ^2^ Hematology and Stem Cells Laboratory, Health Sciences Department, University of Brasília, Brasilia, Brazil

**Keywords:** stem cells, COVID-19, SARS-CoV-2, advanced therapies, exosomes

## Introduction

According to the WHO, approximately 120 million cases of COVID-19 have been confirmed worldwide, with almost 3 million deaths recorded on April 8th, 2021 [https://covid19.who.int/]. Clinically, COVID-19 ranges from an asymptomatic infection to a severe illness, in which patients require mechanical ventilation and hospitalization. Case fatality rates range from <1% up to 20%, and are heavily influenced by patient age, comorbidities [https://ourworldindata.org], as well as geographic location [https://coronavirus.jhu.edu/data/mortality]. In severe and critical patients older than 75 years, case fatality reaches 79.2% (1). Poor outcome patients frequently present progressive pulmonary infiltrates and hyperinflammatory response (2), which are targets of different experimental treatment strategies.

So far, there is no consensus on the standard treatment for critical COVID-19 patients. An overwhelming number of parallel developments are currently underway with this objective, including the administration of Mesenchymal Stem Cells (MSCs) and MSC-derived products, such as exosomes. The low levels of ACE-2 and TMPRSS2 on the MSC surface, the refractory profile of these cells when challenged with SARS-CoV-2 (3), and the successful use of MSCs to treat inflammatory disorders justify the investigation of MSCs and their products for COVID-19 management.

In July 2020, we provided a comprehensive analysis of the clinical trials underway worldwide investigating the application of stem cells and derived products to treat COVID-19 (4). At the time, 69 clinical trials had been initiated, but only 3 scientific publications and 3 company press release documents had been published reporting achieved results.

During the last 8 months, 40 new clinical trials involving MSC-based treatments for COVID-19 were registered, and 8 scientific publications have been published. Such an update constitutes an important addition to the previous comprehensive review, bringing additional data on more than 160 COVID-19 patients which have successfully received MSC and MSC-derived products. Therefore, in this general commentary, we provide an updated analysis of the current evidence regarding the use of stem cells and derived products for COVID-19 treatment with the aim of filling the present knowledge gap regarding the robust demonstration of the feasibility, safety and possible efficacy of such therapeutic alternatives for COVID-19 management.

## New Evidences Regarding the Use of Stem Cells and Derived Products

Among the 8 scientific publications detected by our group during the last 8 months (**Table 1**), 7 report clinical results of MSC-therapy for COVID-19, and 1 reports the use of MSC-derived exosomes. Together, those recent studies describe the treatment of 162 COVID-19 patients, constituting a more robust body of evidence supporting the safety and efficacy profile of MSCs and their products, especially for critical patients.

**Table 1:** Results of stem cell treatments for COVID-19 since June 2020.

Clinical trial identifier and characteristics	Intervention	Cell dose, administration route and other details	Results summary	Ref
**Not informed** - Interventional, randomized, non-blinded	ExoFlo™	Single dose (15ml), intravenous	No adverse events observed within 72 h of ExoFlo administration. A survival rate of 83% was observed. In total, 17 of 24 (71%) patients recovered, 3 of 24 (13%) patients remained critically ill though stable, and 4 of 24 (16%) patients expired for reasons unrelated to the treatment. Patients’ clinical status and oxygenation improved. Significant improvements in absolute neutrophil count and lymphopenia. Mean C-reactive protein, ferritin, and D-dimer reduction of 77%, 43% and 42%, respectively.	(5)
**NCT04252118 - Phase 1** Interventional, randomized, double-blind, placebo-controlled, parallel assignment	UC-MSCs	Three doses of 3 × 10^7^ cells per infusion, intravenous	No serious adverse events were observed. Mechanical ventilation was required in 1 of 9 patients in the treatment group compared with 4 of 9 in the control group. All patients recovered and were discharged. UC-MSCs-treated patients presented a reduced trend in the levels of inflammatory cytokines.	(6)
**ChiCTR2000031494 - Phase 1** Randomized, open-label	UC-MSCs	Single dose of 2 × 10^6^ cells/kg intravenous	In the 28-day mortality rate were 0 of 12 patients in the hUC-MSC treatment group, while 4 of 29 patients in the control group deteriorated to critical condition and received invasive ventilation; 3 of them died. CRP and IL-6 levels were significantly lower from day 3 of infusion, the time for the lymphocyte count to return to the normal range was significantly faster, and lung inflammation absorption was significantly shorter on CT imaging in the hUC-MSC group than in the control group.	(7)
**IRCT20200217046526N2 - Phase 2/3** Controlled, randomized	UC-MSCs and PL-MSCs	Three infusions (200 × 10^6^ cells), intravenous	Significant reductions in serum levels of TNF-α, IL-8 and C-reactive protein (CRP) were seen in all six survivors (a total of 11 patients received MSCs infusion). IL-6 levels decreased in five patients and IFN-γ levels decreased in four patients. Four patients who had signs of multi-organ failure or sepsis died in 5–19 days after the first MSC infusion. All 6 survivors were well with no complaints of dyspnea on day 60 post-infusion. Radiological parameters of the lung computed tomography (CT) scans showed remarkable signs of recovery.	(8)
**NCT04355728 - Phase 1/2** Randomized, controlled, double-blind	UC-MSCs	Two doses of 100 ± 20 X10^6^ cells, intravenous	No serious adverse events (SAEs) were observed in the 12 patients who received UC-MSC infusions. UC-MSC infusions were found to be safe. Inflammatory cytokines were significantly decreased in UC-MSC-treated subjects on day 6. Treatment was associated with significantly improved patient survival, SAE-free survival, and lower time to recovery.	(9)
**NCT04288102 - Phase 2** Randomized, double-blind, placebo-controlled	UC-MSCs	Three doses of 4 × 10^7^, intravenous	A total of 65 patients received MSCs infusion. UC-MSCs administration exerted improvement in whole lung lesion volume compared with placebo. The 6-minute walk test showed an increased distance in patients treated with UC-MSCs. The incidence of adverse events was similar in the two experimental groups.	(10)
**NCT04269525 - Phase 2** Single Group Assignment	UC-MSCs	Four doses, total of 1 × 10^8^ cells, intravenous	A total of 16 patients received MSCs infusions. There were no infusion-related or allergic reactions. The oxygenation index was improved after cell transplantation. The mortality of enrolled patients was 6.25%, whereas the historical mortality rate was 45.4%. The level of cytokines estimated varied in the normal range, the radiological presentations (ground glass opacity) were improved, the lymphocyte count and lymphocyte subsets (CD4+ T cells, CD8+ T cells and NK cells) count showed recovery after cell therapy.	(11)
**Not informed -** Single Group Assignment	AT-MSCs	Three doses of 0.98 X 10^6^ cells/kg, intravenous	Clinical improvement was observed in 9 of 13 patients. No adverse events were related to cell therapy. Seven patients were extubated and discharged from ICU while four patients remained intubated. Two patients died (one due to gastrointestinal bleeding unrelated to MSC therapy). Treatment with AT-MSC was followed by a reduction in C-reactive protein, IL-6, ferritin, LDH and D-dimer.	(12)

MSCs, Mesenchymal Stem Cells; AT-MSCs, Adipose Mesenchymal Stem Cells; PL-MSCs, Placental Mesenchymal Stem Cells; UC-MSCs, Umbilical cord Mesenchymal Stem Cells.

### Study Characteristics

In our original study published in July 2020, we identified 13 phase 1, 13 phase 2, 19 phase 1/2, 1 phase 2/3 and 1 phase 3 study. Since then, the clinical phase of published studies is now more advanced and includes the publication of a primary report of a 2/3 study. Nevertheless, most of the newly identified trials are still in phase 1 and phase 2 studies, and the information regarding 6 out of 40 trials was not clear.

Most clinical studies underway consist of randomized trials. Most frequently, the control groups include placebo and routine treatment. Some studies still lack control groups, and 1 study is a single center, retrospective investigation.

Cell-based therapy remains the most explored therapeutic strategy. While 38 studies intend to investigate the use of MSCs to treat COVID-19, two studies focus on the use of MSC-derived products (NCT04602442 and NCT04753476). Thirty-four will administer the cells intravenously. One study proposed the inhalatory route for exosome treatment, and one study proposed the intramuscular route for MSC-secretome administration. The proposed number of infused cells was similar to previously detected, ranging between 0.5x10^6^ and 3x10^8^ cells, with the most frequently proposed dose consisting of single or multiple infusions of 1x10^6^ cells/kg.

In the MSC source side, the umbilical cord was the most commonly chosen MSC source (27%), as previously noticed, followed by the bone marrow (20%), adipose tissue (15%), dental pulp (7.5%), Wharton’s jelly (5%), placenta and amniotic membrane (1 study each).

Considering published results, the 8 new scientific publications detected describe the data obtained from 160 patients which received MSC-based advanced therapies for COVID-19 management. Different from the scarce safety and efficacy evidence observed in July 2020, the current evidence supporting the notion that MSC and MSC-derived therapeutic products are safe and effective are based on the reports of several different research groups distributed worldwide. The most prominent beneficial effects of MSC and MSC-derived therapeutic products include the improvement of patient breathing capacity, the mitigation of cytokine storms, the restoration of the immune system, the decrease of hospitalization time, and the increase in patient survival rates.

## Discussion

The COVID-19 pandemic has provoked a rapid mobilization of the scientific community in the attempt of providing control, prevention, and treatment strategies for COVID-19. In the absence of a gold-standard treatment for the disease (13), it is important for physicians to keep the pace regarding the real evidence supporting different experimental treatment strategies. For instance, as reported by Servick et al., in the last few months, “physicians memorized treatment guidelines one day only to learn they’d changed the next” (14).

Since July 2020, important data have been published demonstrating that the use of MSCs for severe COVID-19 treatment is increasingly promising. In the last 8 months, more than 160 patients received MSC and MSC-derived therapies. In contrast to the 51 patients treated with MSC-based advanced therapies for COVID-19 between January and July 2020, current evidence is now based on the data obtained from more than 200 COVID-19 patients. Importantly, the published data available at this point clearly indicates that the infusion of MSCs is feasible, safe, and that it possibly constitutes a highly effective treatment option for severe cases of COVID-19. Evidently, a final conclusion regarding the effects of MSCs administration and its products on the mortality of patients with COVID-19 depends on the complete data of large, randomized phase 3 trials, which are eagerly awaited (14).

Possibly due to the successful data obtained at this point, no important differences were detected considering the clinical trial design, MSC source, dose, and administration route proposed by the latest clinical studies registered in clinical trial databases.

As discussed in our first manuscript, the proposal of treating COVID-19 patients with MSC and MSC-products derive from the partial knowledge regarding the COVID-19 pathophysiology, but also from previous experience in the treatment of other respiratory diseases, such as influenza. Therefore, it is important to acknowledge the importance of accumulating clinical experience in the use of MSCs, in order to rapidly expand its application in times of need. It is indeed possible that the immunization of the world’s population will constitute the main strategy to prevent COVID-19 spread, and that the current treatment alternatives based on dexamethasone, Remdesivir, and monoclonal antibodies (13–15), will possibly decrease the need for advanced therapies for COVID-19.

Still, it is important to acknowledge that, especially for critically ill patients, COVID-19 importantly affects multiple organs (16), in such an extent that these patients are beyond the point where antiviral alternatives might restore organismal function. In this scenario, the pleiotropic and systemic anti-inflammatory and regenerative effects of stem cells may greatly benefit severe patients, justifying further investigation (17). Regardless of stem cell treatment becoming the gold standard for severe COVID-19, the lessons learned by the regenerative medicine research community in the short period of the last 15 months will be forever useful for future therapeutic applications of such products.

## Author Contributions

JC, AS-C, EM, and FS-A researched data and wrote the manuscript. All authors contributed to the article and approved the submitted version.

## Funding

This work was supported by CNPq (National Council of Technological and Scientific Development), CAPES (Coordination for the Improvement of Higher Education Personnel), and FAPDF (Federal District Research Foundation).

## Conflict of Interest

The authors declare that the research was conducted in the absence of any commercial or financial relationships that could be construed as a potential conflict of interest.
